# Twists and turns: The whirl sign in a patient with systemic sclerosis

**DOI:** 10.2478/rir-2024-0025

**Published:** 2024-10-21

**Authors:** Lin Qiao, Dong Xu

**Affiliations:** Department of Rheumatology and Clinical Immunology, Chinese Academy of Medical Sciences & Peking Union Medical College; National Clinical Research Center for Dermatologic and Immunologic Disease (NCRC-DID), Ministry of Science & Technology; State Key Laboratory of Complex Severe and Rare Diseases, Peking Union Medical College Hospital (PUMCH); Key Laboratory of Rheumatology and Clinical Immunology, Ministry of Education, Beijing 100730, China

**Figure 1:**
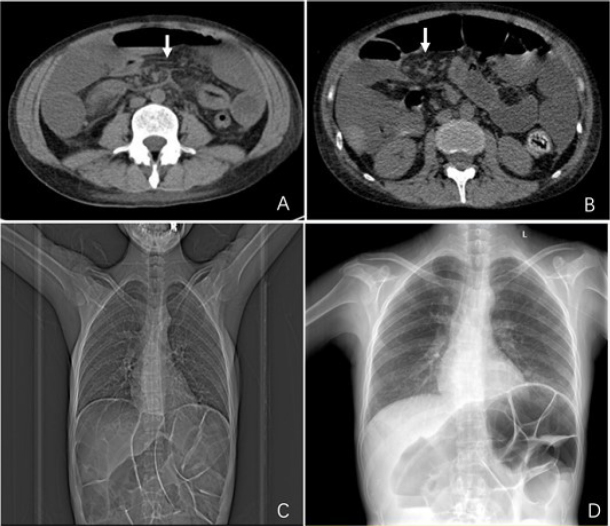
Computed tomography scan of the abdominal. The whirl sign (arrow) under abdominal X-ray (A and B) before treatment (C) and six months after treatment (D).

A 36-year-old female was admitted because of progressive skin thickening for 9-years. During the past9 years, she experienced Raynaud’s phenomenon, facial and extremity skin thickening, exertional dyspnea, abdominal distension, and muscle pain along with time. Her serologic investigation revealed a positive anti-nuclear antibody with a titre of 1: 80 in a homogeneous pattern. The patient was diagnosed as having limited systemic sclerosis (SSc) with lung and gastrointestinal involvement. Low-dose glucocorticoid combined with azathioprine were initiated. She had intermittent diarrhea with abdominal distension in the past 4 months and she was diagnosed as pseudo-obstruction (IPO). Ultrasound examination revealed thickened small intestine wall with evident intestinal dilation. A subsequent abdominal computed tomography (CT) scan revealed dilated fluid-filled bowel loops, with thickened bowel wall and an unusual mesenteric presentation. The mesentery was twisted and there was diffuse engorgement of the mesenteric vessels along with diffuse haziness. CT revealed a whirl sign of mesentery and wrapped small bowel loops (Figure 1). She was treated with fasting and total parenteral nutrition were initiated to improve her nutrition. She was temporarily started on oral prednisolone (20 mg/d), intravenous cyclophosphamide (0.4 g/week), monthly immunoglobulin (400 mg/kg over 3 days) and probiotics. An improvement in bowel motility was observed. Abdominal X-ray image demonstrated a decrease in intestinal gas. Then she resumed semiliquid diets gradually, and received six courses (400 mg/kg over 3 days) of immunoglobulin infusion, and her glucocortoid were tapered. Cyclophosphamide infusion was withdrawn due to severe thrombocytopenia. Six months later, her abdominal symptoms and muscle pain improved, and her body weight increased from 38 kg to 42 kg.

Patients with SSc can experience a variety of gastrointestinal dysmotility starting from the esophagus to the anorectum.^[1,2]^ In patients with SSc, chronic IPO, which is characterized by clinical and radiological evidence of chronic episodic intestinal obstruction in the absence of a mechanical lesion, presents as a dilated and atonic small bowel with delayed transit. The whirl sign, which is characterized with a spiraled loop of collapsed bowel and engorged mesenteric vessels on a CT scan is an important sign for the diagnosis of volvulus. However, there is no evidence that showed the effectiveness of immunosuppressive therapy in this condition. Previous investigations^[3,4]^ suggested that immunoglobulin infusion might be beneficial. In our case, her condition was improved after being treated with immunoglobulin infusion and control of oral intake.
